# Clinically relevant phenotypes in chronic rhinosinusitis

**DOI:** 10.1186/s40463-019-0350-y

**Published:** 2019-05-29

**Authors:** Jessica W. Grayson, Marina Cavada, Richard J. Harvey

**Affiliations:** 1 Rhinology and Skull Base Research Group, St Vincent’s Centre for Applied Medical Research, University of New South Wales, 67 Burton Street, Darlinghurst, Sydney, NSW 2010 Australia; 2 Department of Otolaryngology, Faculty of Medicine and Health Sciences, Macquarie University, Sydney, Australia

## Abstract

**Background:**

Chronic rhinosinusitis (CRS) is a complex disease that incorporates many different conditions. Currently, primary CRS is considered a disease of broad airway inflammation, however, the previous classification of CRS with and without nasal polyposis fails to adequately classify patients based upon their etiology of illness. Our aim with this review is discuss the clinical presentation, radiology, endoscopy, histopathology, and treatment algorithm of three different phenotypes of primary CRS: central compartment atopic disease, eosinophilic CRS, and non-eosinophilic CRS.

**Methods:**

A narrative review of a tertiary rhinology center’s research themes and how they are applied to clinical protocols and practice was assessed.

**Discussion:**

Diagnosis and treatment of upper and lower airway conditions become increasingly important as phenotypes and endotypes are being described. There are well-described therapies to treat the different phenotypes of CRS, based upon the presumed underlying cause of the inflammatory process. Research continues to shed more light on different endotypes and phenotypes of airway inflammation, however, clinical differentiation of CRS can be applied in clinic practice with three simple phenotypes of CRS. Understanding these different phenotypes and their etiologies allows for further management beyond the ‘maximum medical therapy and then surgery’ approach that has often been used in the management of CRS.

## Introduction

Chronic rhinosinusitis is a complex disease that has previously been used to describe conditions ranging from unilateral single sinus disease, odontogenic sinusitis, fungal sinusitis, to widespread airway inflammation. The currently recognized definition of primary CRS is represented by chronic inflammation of the paranasal sinuses. In this discussion, primary CRS refers to a sinus condition in which no obvious secondary pathoetiologic event is occurring (ie fungal ball, neoplasia, odontogenic or immunodeficiency). Classically, primary CRS has been separated into two major subtypes based upon phenotypic appearance; CRS with nasal polyps (CRSwNP) and CRS without nasal polyps (CRSsNP) [1, 2]. The description of these different subtypes relies largely on observable clinical findings and lacks the inclusion of molecular differentiation and potential molecular diversity that exists within a subtype. Additionally, eosinophilic rhinosinusitis, allergic fungal sinusitis (AFS) and aspirin exacerbated respiratory disease (AERD) have been proposed as subtypes of CRS. In our practice, the treatment paradigm for CRS is largely driven by the presumed underlying etiology and molecular endotype of each inflammatory condition. This is also true for inflammatory disease of the lower respiratory tract, as the upper and lower airway are one unified tract and therefore exhibit similar molecular pathophysiology.

Previous studies on the success of different treatment options in the lower respiratory tract have had varied results and it is likely due to vague clinical definitions and use of only phenotypic descriptions driving treatment decisions [3]. ‘Endotyping’ of both asthma and CRS has been the focal point of more recent studies, as well as categorizing endotypes with their associated phenotypic group [3–5]. This initially started in the evaluation of asthma and has been extrapolated and studied in CRS. This differentiation will allow for more efficient and efficacious treatment of these inflammatory conditions as well as allowing for improved analyzation of the outcomes of treatment.

The traditional CRS phenotype of with and without nasal polyps has significant limitations. We describe three major clinically relevant phenotypes of CRS associated airway inflammation that exist: allergic, eosinophilic, and non-eosinophilic. The purpose of this paper is to define the different clinical and pathologic features of these CRS phenotypes, as well as the treatment options and goals.

## Methods

The St Vincent’s Centre for Applied Medical Research Rhinology and Skull Base Research Group research themes regarding CRS were reviewed. The current clinical protocols at a tertiary rhinologic practice were reviewed for the following criteria: patient selection (presenting symptoms including allergy and/or broader airway involvement, age of onset, smell function, corticosteroid responsiveness), endoscopic evaluation (the location and distribution of polypoid changes, degree of eosinophilic mucin production), radiologic assessment (a central versus diffuse pattern of mucosal changes), histopathology (degree of eosinophilia, evidence of eosinophil activation), atopic or allergen sensitization status (epicutaneous allergen challenge or allergen-specific serum immunocap assessment), and treatment goals (both medical and surgical therapy). The clinical diagnostic and treatment protocols for various working CRS phenotypes are described using the above criteria.

### Phenotype

#### Central compartment atopic disease (CCAD) (allergic (IgE-mediated) airway inflammation)

##### Defining patient characteristics

Patients with allergic airway inflammation often have an earlier onset (< 20 yo) disease, and although there is eosinophilic T-helper 2 (T_H_2) cell involvement, it is predominantly IgE driven with other signs of atopic disease [3]. These patients will likely endorse a history of systemic atopy including symptoms of allergic rhinitis (AR), conjunctival symptoms, and dermatitis [3]. They will also have a history of allergic asthma (asthma associated with childhood and not adult-onset) with similar triggers to their other allergic symptoms. Most patients with persistent asthma that had an onset in early childhood suffer from allergic airway inflammation [3].

Local symptoms are more dominated by itch, sneeze, and rhinorrhea. Conjunctival reactivity is common. They will often have retained their sense of smell, despite having been told by other practitioners that they had nasal polyps [6]. The symptoms remain corticosteroid responsive as they are a T_H_2 inflammatory response, but are simply driven by IgE mediated triggers. While the relationship between CRS and allergy has often been studied, only weak evidence supports a connection between the two [7]. However, there is good evidence that IgE driven allergic patients develop a form of CRS on endoscopy and radiology that is unique [8].

##### Endoscopy

**Fig. 1:**
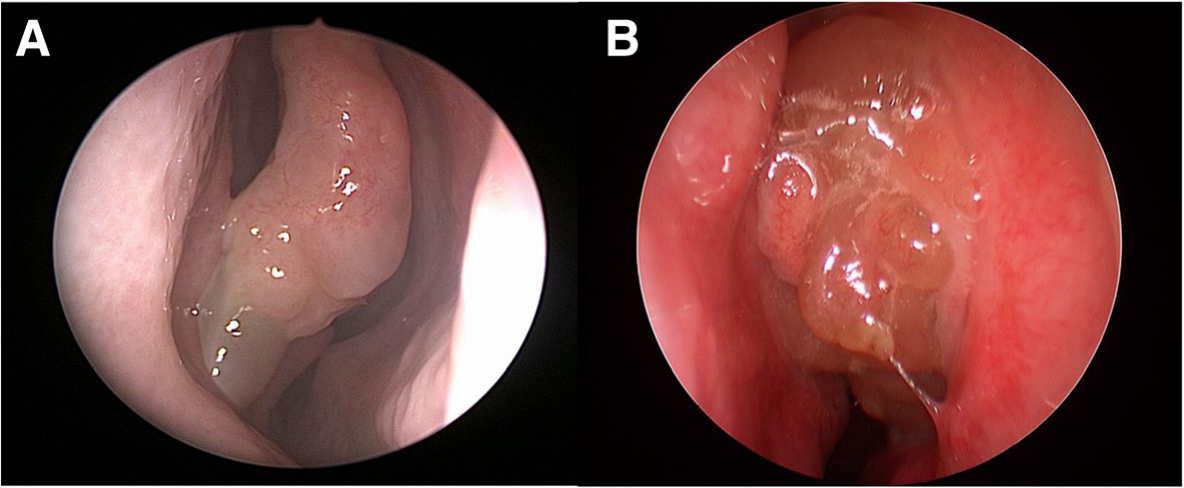
**a** – Middle turbinate edema present in CCAD or IgE drive airway inflammation; **b** – Middle meatus polyposis present in eCRS airway inflammation

Atopy in CRS can manifest as middle turbinate edema, which can be viewed on nasal endoscopy (Fig. 1a). The middle turbinate is representative of ethmoid mucosa without the vascularity and fibrous stroma that is seen in the inferior turbinate. In patients with allergic rhinitis (AR), inhaled allergens are deposited on the head of the middle turbinate, which can lead to inflammation and edema of the mucosa. Hamizan et al. [9], found that patients with diffuse or polypoid edema of the middle turbinate had a higher likelihood of presence of inhalant allergy (PPV = 91.7 and 88.9% LR+ = 8.0 and 6.2). In more severe cases, the middle turbinate edema can progress to involve the superior turbinate and posterior nasal septum and potentially cause obstruction to more lateral sinus ostia [10]. However, these patients will not exhibit the thick eosinophilic mucin that is noted in other inflammatory airway conditions. Subsequently, the cycle of infective or acute exacerbations that are seen in diffuse eCRS is less common in this allergic phenotype.

At the time of surgery, polypoid changes are restricted to the turbinates, uncinate and ostiomeatal complex (OMC) [6]. Despite the extent of polypoid change on these structures, there is often near normal ethmoid, sphenoid and maxillary mucosa. Simple trapped mucus is all that can be found in many patients.

##### Radiology

**Fig. 2:**
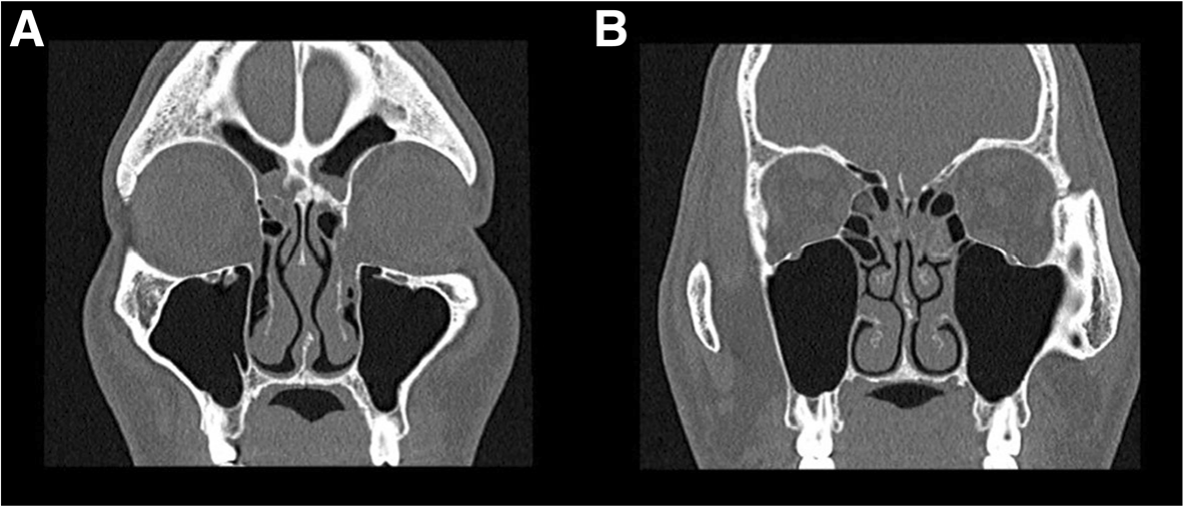
**a** - Radiographic evidence of CCAD with central disease and peripheral clearing; **b** - “black halo”sign

The hallmark of inhalant/IgE driven CRS is a central thickening of the turbinates and septum with near normal peripheral sinus mucosa. This was originally described by Lund et al. as the “black halo” sign [11]. On computed tomography (CT) these sinus changes will appear as soft-tissue thickening in the central portion of the nasal cavity with sparing of the roof and lateral walls of the sinus cavity [8] (Fig. 2). Secondary obstruction can occur when the central changes began to encroach laterally on the sinus outflow tracts. If sinus changes occur, they are often simply secondarily obstructive in nature with trapped mucus.

##### Histopathology

Histopathologically, T_H_2 cytokines dominate this condition. This leads to elevated total and serum specific IgE. These patients will rarely have an elevated serum eosinophil count [3]. Tissue eosinophils are identified on simple hematoxylin and eosin (H&E) stain, and at this stage a further discriminated appearance is not available. However, there is rarely eosinophil aggregates or charcot-leyden crystals on mucin examination, as these are features of activated or degranulating eosinophils.

##### Allergy testing

Patients who suffer from allergic airway inflammation have higher serum specific IgE when compared to those who are suffering from other subtypes of asthma [3]. These patients will have evidence of atopy on skin prick test or Immunocap/radioallergosorbent test (RAST) and are associated with clinically relevant triggered symptoms. Persistent allergen exposure and inflammation is normally required to produce this phenotype therefore, seasonal allergens are not normally the cause of this type of CRS as chronic activation is usually requires in the authors experience. Dustmite and other perennial allergens are more likely.

##### Treatment goals

While surgery is still often applied to remove polypoid changes and prevent secondary obstructive phenomena that have occurred, it is the underlying inhalant allergy that needs to be suppressed in order for symptoms to improve. It is not uncommon to see patients post-surgery equally as symptomatic with normal appearance to their sinus cavity and progressive polypoid changes on the residual turbinates. If the polypoid changes have occurred over a long period of time and the underlying allergic process is indolent, then surgery may be the first step to overcome nasal obstruction and other phenomena such as barotrauma [10]. Once polypoid changes have occurred simple corticosteroid sprays are unlikely resolve the remodeling that has already occurred. Allergy directed pharmacotherapy and immunotherapy can occur post-surgery.

However, if the symptoms are dominated by an active allergic process with reactive nasal and conjunctival symptoms, then immunotherapy should be considered first and surgery delayed until a less allergic and more stable airway mucosa is obtained.

Only in those who cannot tolerate immunotherapy, have hyper-IgE states, and/or persistent allergy that has failed immunotherapy/pharmacotherapy, we do consider additional therapies such as Omaluzimab (anti IgE).

#### Eosinophilic airway inflammation

##### Defining patient characteristics

Eosinophilic CRS (eCRS) is an inflammatory condition due to a T_H_2 responses that is driven by eosinophilic inflammation. These patients tend to be older (30-50yo at onset) compared to those with early onset allergic disease (< 20 yrs. and *“I’ve had sinus all my life*” patients). Another common presenting pathway is an adult patient who was *“completely well”* until they had a viral event or cold and then *“never recovered”.*

Although some of these “adult-onset” eCRS patients may have had a history of allergic disease in their childhood, they often report that it quiesced for many years, with few symptoms in their early adult life only “*to come back”* in their 30-50 yrs. When there is a history of allergy, there is a strong association bias to believe it is related to their CRS, however 20–30% of the population have some allergic or atopic predisposition. Any adult onset eCRS condition with a history of allergy that had previously become quiescent or burnt-out, is likely to be unrelated to their childhood atopy.

Smell loss is a major feature early in the disease process for eCRS sufferers. Acute exacerbations, likely due to secondary obstructive or infective phenomena are common and these adult onset eCRS often seek antibiotics on multiple occasions prior to presentation [12]. Corticosteroid responsive smell or olfactory recovery is another common feature.

Patients almost always report a history of asthma at around the same time as the onset of their upper airway symptoms. In those patients who do not present with asthma, they will likely become asthmatic. Variants of lower airway disease include unexplained but corticosteroid sensitive cough, and obvious wheeze in a patient who lacks the classic bronchodilator criteria for asthma (GINA criteria).

eCRS patients will often report food induced and alcohol related exacerbations or flares of their condition. This is thought to be related to eosinophilic priming of the mucosa and leukotriene induced trigger. Aspirin exacerbated airway disease, includes non-steroidal anti-inflammatory (NSAIDs) reactivity, is a subtype of adult-onset eCRS.

Corticosteroid responsiveness is major feature of these patients. They often report a dramatic benefit within days of starting therapy and smell often returns quickly. Many patients seek corticosteroid and it is not uncommon to have patients wanting to be on them or self-medicating, although most are familiar with their side-effects. Patients who would report that corticosteroids are a *‘magic pill’* are often eCRS sufferers. This response is due to the penetration of corticosteroid to the sinuses, which occurs systemically via the blood stream in the unoperated sinus cavity. Simple corticosteroid sprays have little effect in most patients [13].

##### Endoscopy

Endoscopically, these patients will have evidence of thick tenacious eosinophilic mucin. They can also have polypoid edema, many small polyps, or true large polyps in the nasal cavity (Fig. 1b). During surgery, mucinous casts and “chewing gum” like mucin is classically present with diffusely thickened mucosa throughout the sinus cavity. Acute mucosa edema and ‘peau dórange’ appearance is more common in adult eCRS, especially in the early phase of the condition. The polypoid changes arise from within the middle meatus and are simply redundant changes from the turbinates as in allergy based CCAD.

##### Radiology

**Fig. 3:**
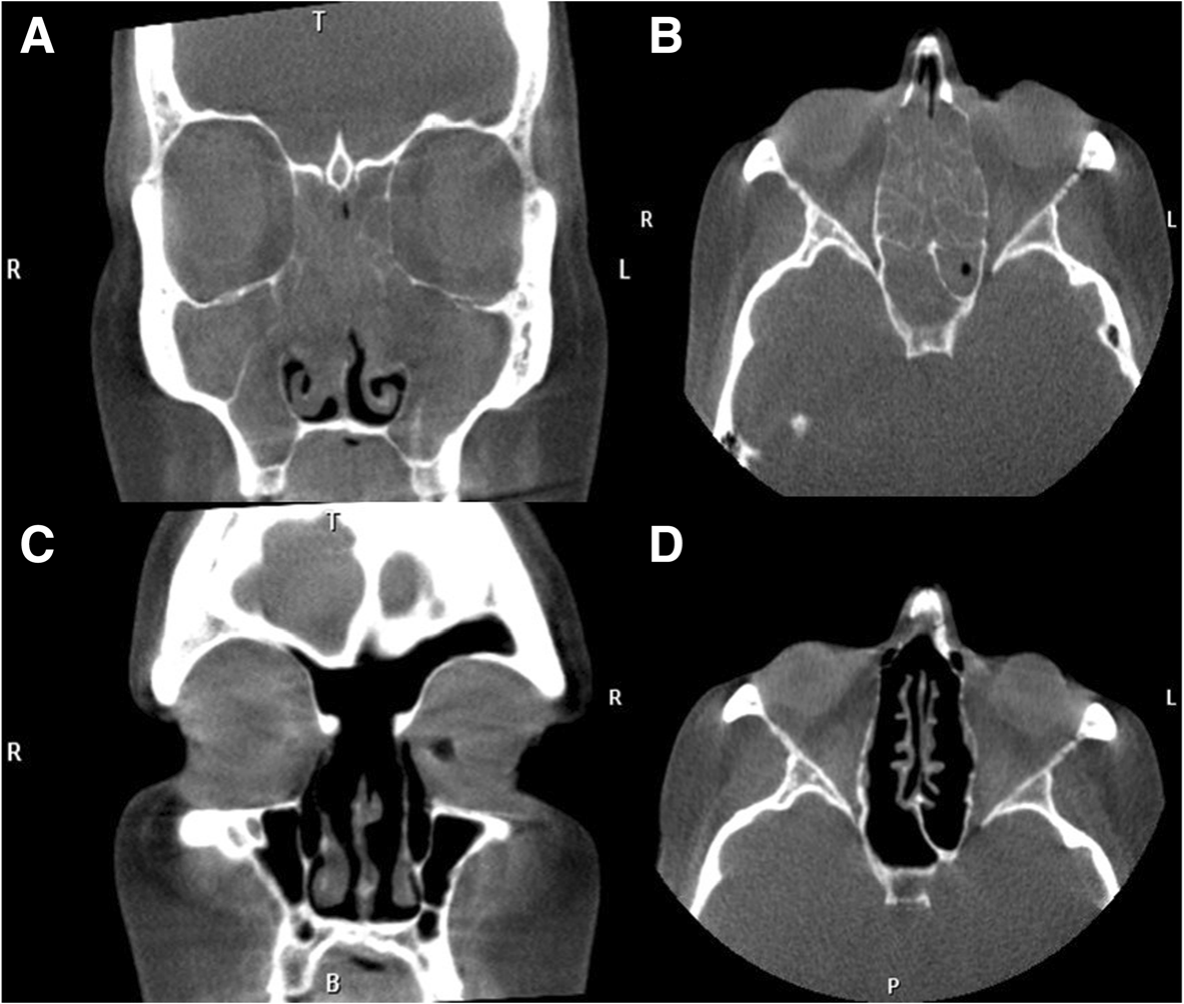
**a**,**b**: Pre-operative CT images view of patient suffering from eCRS; **c**,**d**: Post-operative CT images of patient compliant on corticosteroid irrigations

These patients have classic pan-sinus opacification with a Lund-Mackay score of 24. They may have evidence of secondary obstruction present on radiographic imaging (Fig. 3a-d). Neo-osteogenesis changes are common even in the un-operated patient.

##### Histopathology

**Fig. 4:**
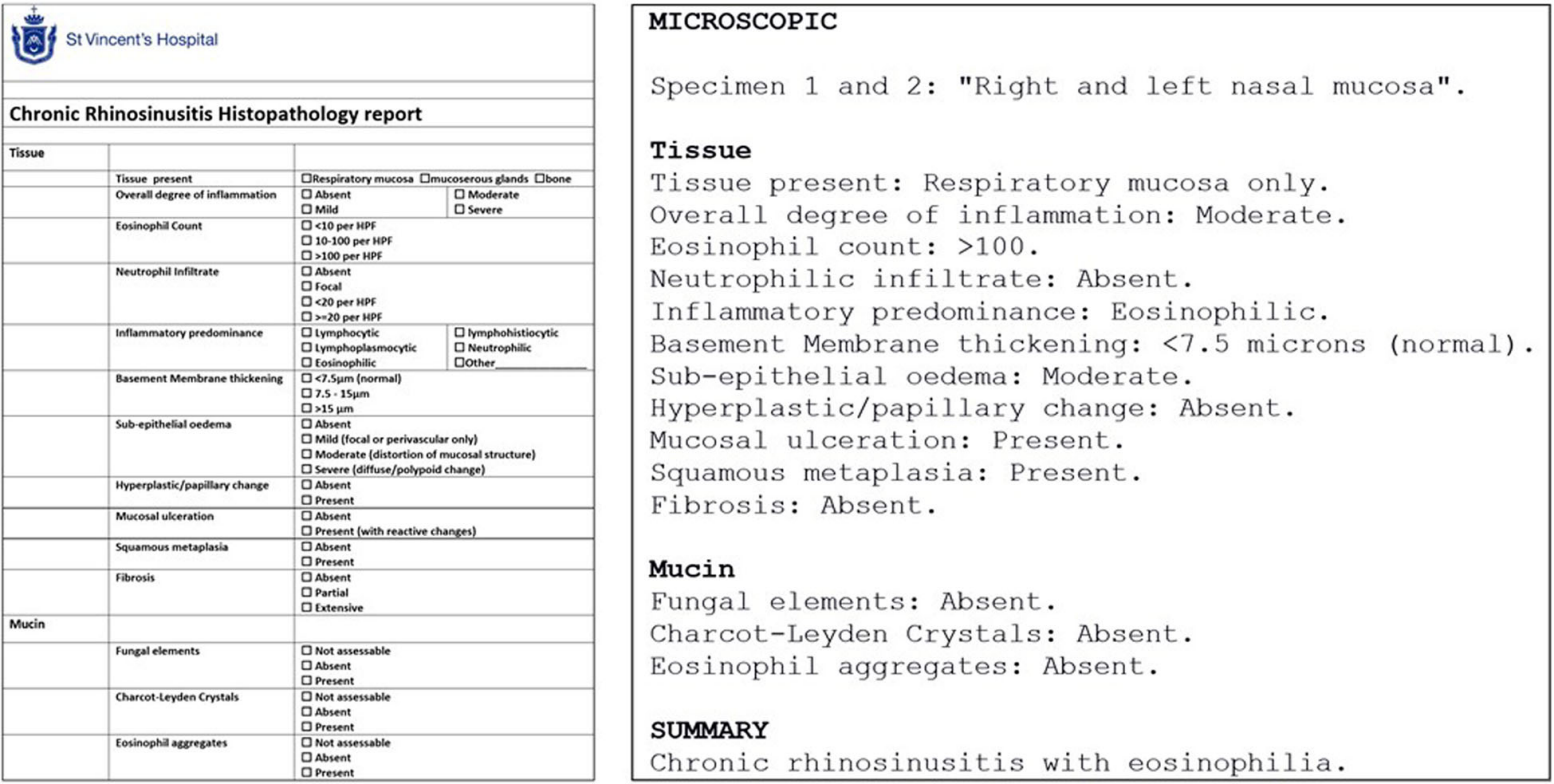
Sample histopathology report

Histopathologically, these patients will have tissue eosinophilia. Greater than 10 eosinophils/high powered field (hpf) is considered the current definition, however, it doesn’t distinguish between CCAD and eCRS. Many patients have sinus biopsies with > 100 eosinophils/hpf (Fig. 4). A recent systemic review has demonstrated that a cut off 55 eosinophils/hpf predicts the likelihood of recurrence following surgical intervention [14].

Systemic eosinophilia is a feature of eCRS. High levels of eosinophils in the blood have a positive likelihood ratio (LR) of 3.28 to predict high tissue eosinophilia [12]. Tissue eosinophilia, however, is not significantly associated with serum allergen specific IgE [12].

##### Allergy testing

These patients will have either, absolutely negative IgE sensitization or a pan or multi allergen sensitivity. There are mechanisms that induce local mucosal IgE generation that can produce multi-allergen sensitivity [15].

##### Treatment goals

Therapy should be aimed at treating what is primarily an inflammatory disorder. The goal of therapy is to deliver anti-inflammatory medicine to the site of the disease with the least amount of side effects or systemic exposure. In mild cases, associated with exacerbations and limited burden of disease, intermittent short courses (2–3 weeks) of corticosteroids can be offered 2–3 times per year. If systemic treatment is needed more often than this on an ongoing basis, then the risks of corticosteroid related adverse events in significant [16].

**Fig. 5:**
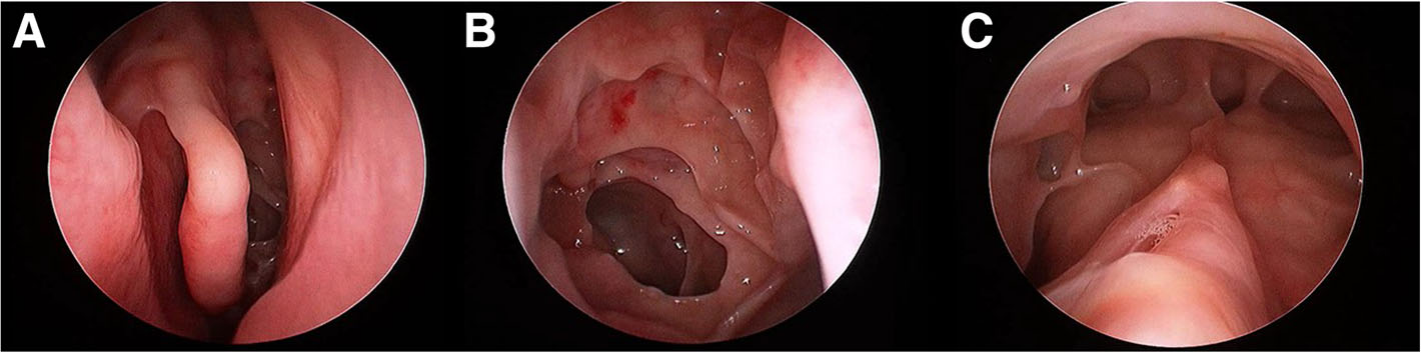
Widely opened sinus cavity view of **a**: middle meatus, **b**: sphenoethmoidectomy, **c**: draf 3/Lothrop cavity

Effectively delivered corticosteroid solution via nasal irrigation is the key to controlling adult onset eCRS long-term. At some point, many patients will need to consider shifting their anti-inflammatory treatment from intermittent systemic delivery to regular local topically delivered corticosteroid. It has been well recognized that the delivery device and the surgical state of the sinus cavity effect the ability of local therapies to access the sinus cavity. Large volume (240 mL) irrigations achieve the best distribution when used in a simple sinus cavity [17, 18]. In a double blind, randomized controlled trial, it was noted that corticosteroid irrigation provided better overall symptom control that corticosteroid nasal spray [18]. Studies have also shown that wide openings in the frontal [19], maxillary [20], and sphenoid [21] sinuses allow for improved irrigant penetration and lavage of the sinus cavity (Fig. 5). Typical corticosteroid solutions include budesonide 1mg [22], mometasone (2 mg) [18], or betamethasone (1 mg) [22, 23] in a 240 mL irrigation once a day.

Even with excellent topical therapy via an adequate surgical cavity and large volume corticosteroid irrigations, some patients may not fully suppress their eosinophilic airway inflammation. Just as in asthma management, not all inflammatory airway patients are controlled with inhaled medications alone. Rescue medication with short courses of corticosteroid may still be required, especially with viral induced exacerbations. Mepolizumab is an anti-IL5 monoclonal antibody that is currently used in uncontrolled eosinophilic asthma and most of these patients have uncontrolled eosinophilic sinus disease. These patients’ eCRS condition does very well from the introduction of anti-IL5 therapy to their care. There are other monoclonal antibody treatments, targeting tissue eosinophilic response Benraluzimab (anti-IL5 receptor), Dupilumab (anti-IL4 receptor alpha subunit/IL13), and Reslizumab (anti-IL5) have been used in the treatment of eosinophilic asthma and related eCRS [24]. Prognostic features of patients with eCRS, who are at risk of failing a topical therapy approach, include those that have lower airway disease that is not well controlled on preventative inhaled therapy and/or require systemic corticosteroid therapies to control lower airway symptoms. In these patients, there is a realistic expectation that their upper airway will behave similarly.

Patients who suffer from the AERD subtype undergo the same medical therapy as those with adult-onset eCRS. The use of 5-lipoxygenase antagonist (Zileuton) and/or leukotriene receptor antagonist (Montelukast and Zafirlukast) may assist these patients. It is important to note that the 5-lipoxygenase antagonist and leukotriene receptor antagonist do not prevent symptoms in patients undergoing aspirin desensitization. These patients can take selective COX2 (cyclooxygenase) inhibitors (Meloxicam, Celecoxib) without risk of cross-over inhibition of COX1 [25]. In these patients, as well as patients who are unable to avoid NSAIDs for medical necessity, aspirin desensitization can be considered to the treatment paradigm [25]. Aspirin desensitization can occur in an inpatient or outpatient setting and typically is initiated over 2–3 days. Aspirin challenge dosing is 20-40 mg and increases by 20-40 mg every 3 h. Typical commencement protocol is 1300 mg daily (650 mg twice daily) for one month, if there is improvement in symptoms, then the dosage can be decreased by 350 mg. Maintenance doses should maintain 350 mg once or twice daily [25].

#### Non-eosinophilic airway inflammation

##### Defining patient characteristics

Unlike eosinophilic and allergic airway inflammation, patients with non-eosinophilic airway inflammation tend to be middle aged (~60s), female, obese, and have no significant history of atopic disease. These patients will have a history of broader airway inflammation that is often not completely controlled on inhaled corticosteroids. If they have a history of childhood atopy, they will no longer suffer from classic allergic symptoms.

They will less often have smell loss. However, when they do, corticosteroids will not be the *“magic pill*” that they are for eCRS patients. Even with adequate treatment and control, their sense of smell will be slower to return. If patients have been treated in the past with corticosteroids, they may report very mild to no improvement. These patients are often on classic preventative inhaled therapy for their lower airways symptoms and still have break through symptoms.

##### Endoscopy

**Fig. 6:**
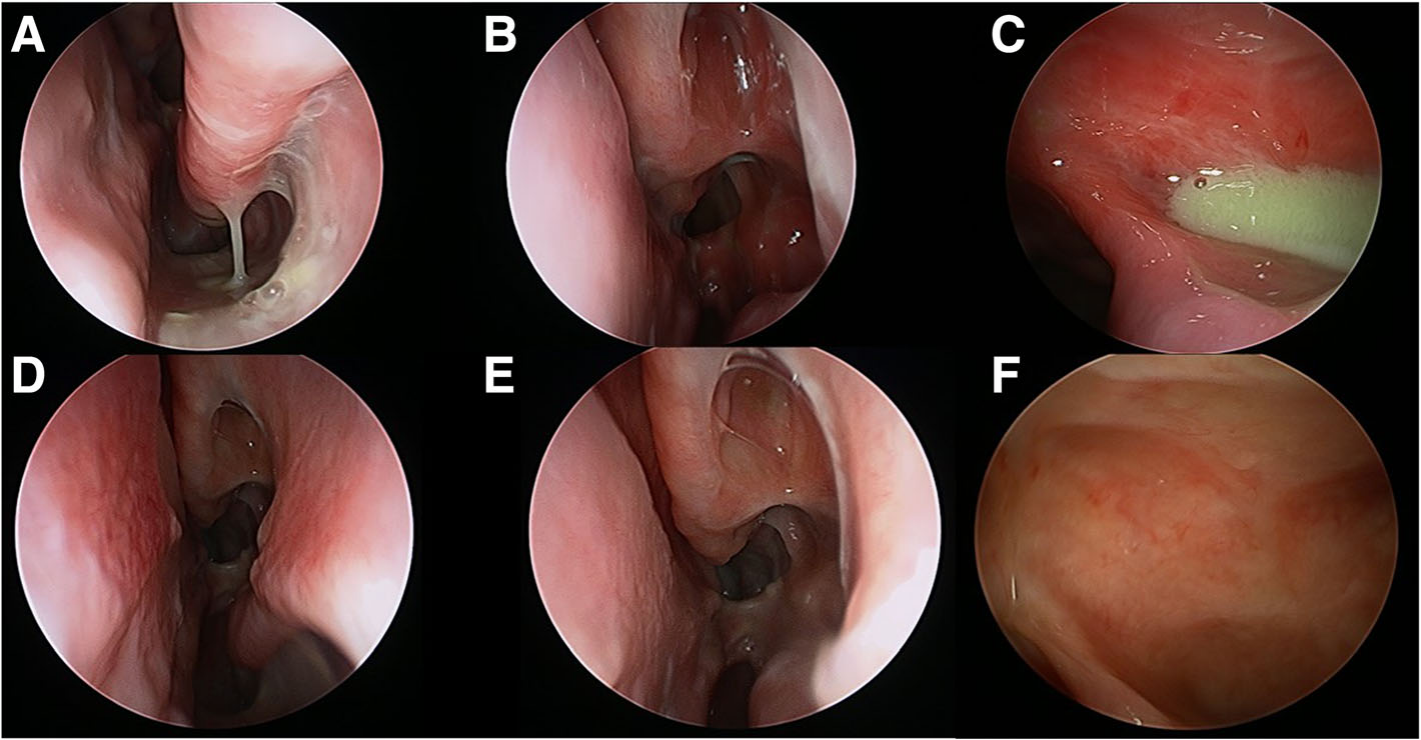
**a**-**c**: Endoscopy images of a 70 yo male patient with non-eCRS referred for revision surgery, although some synechiae had formed, he had limited response to corticosteroid with diffuse airway symptoms suffering from non-eCRS following sinus surgery, but with persistent symptoms; **d**-**f**: Endoscopy images of same patient 3 months after initiation of macrolide therapy (Clarithromycin 250 mg daily)

Nasal polyps or polypoid edema can be seen in these patients. They will not have eosinophilic mucin. However, they will have thick discolored secretions and obstruction of sinus outflow tracts which can lead to post-obstructive infective phenomena and purulence (Fig. 6).

##### Radiology

**Fig. 7:**
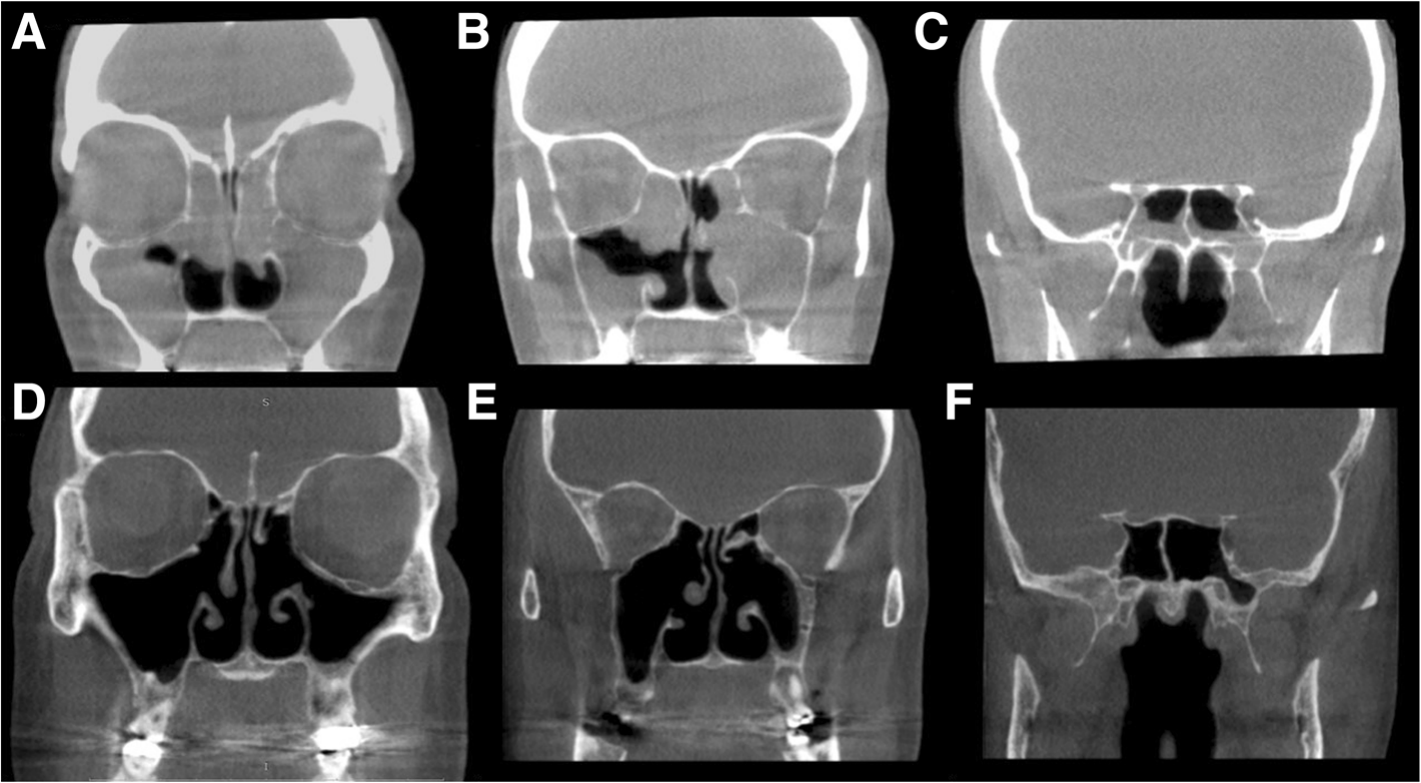
CT images of patient suffering from non-eCRS airway inflammation compliant on topical therapy; **a**-**c**: Post-operative imaging, with persistent disease; **d**-**f**: After initiation of macrolide therapy for 3 months (Clarithromycin 250 mg daily)

The patients are often indistinguishable from the pan-sinus opacification seen in eCRS with high Lund-Mackay scores (Fig. 7a-c). Importantly, this is still an inflammatory disorder and the sinuses are diffusely involved.

##### Histopathology

Inflammation is driven by neutrophils and tissue eosinophils will be low. Tissue neutrophilia is significantly correlated with the presence of pro-inflammatory cytokines (IL-1β, 6, and 8) [26, 27]. Increasing levels of the non-Th2 cytokines present in the mucus is correlated with higher culture positivity and age [27].

##### Allergy testing

These patients generally will have negative skin prick and immunocap/RAST testing and lack clinical evidence of allergen driven symptoms.

##### Treatment goals

Although these patients do benefit from having sinus surgery, to overcome secondary obstructive phenomenon and mucostasis, the delivery of corticosteroids via nasal irrigation rarely controls the disease much like the poor response to systemic corticosteroid. This non-eCRS phenotype does well from long-term low dose macrolide immunomodulation [28, 29]. Macrolide therapy has been shown in previous studies to mediate IL-8 in nasal secretions, inhibit neutrophil chemotaxis and promote neutrophil degradation in a way that corticosteroids cannot [28]. Given this effect on neutrophils and mediators of neutrophils, Oakley et al. [29], described the patients who would best respond to therapy to be those with low serum and tissue eosinophilia and no presence of squamous metaplasia on histopathology. However, clinically, one could surmise that patients with late onset (>60yo) CRS and with limited response to corticosteroids could potentially benefit from a trial of macrolide therapy. Patients can be initiated on low daily dose of a macrolide (Clarithromycin 250 mg) and re-evaluated for benefit after 3 months. Those who receive benefit can be tapered to 3 doses per week and treatment can be ceased after 1 year, with expected lasting benefit (Fig. 6d-f, 7d-f). In our practice, only patients with a biopsy proven phenotype are offered this treatment, and thus only a post-surgical treatment. This is also practical as these patients can also be screened for prolonged QT syndrome [30].

## Conclusion

The philosophical approach outlined in this publication represent the working classification of a tertiary center that has moved away from a simple CRSwNP and CRSsNP classification. There is no doubt that this paradigm will change with advances in research and understanding of CRS. However, the profession has been slow to move away from the current dichotomous simple CRS classification despite the wealth of research and data that has evolved in the past decade.

Diagnosis and treatment of upper and lower airway diseases becomes increasingly important as several different phenotypes are described. There is well-defined therapy to the presumed underlying nature of the inflammatory disease process. While research continues to define both phenotypes and endotypes, the clinical differentiation of CRS can be applied to daily practice with these three simple CRS phenotypes. Although this approach will evolve with our understanding of the disease and the emergence of new biomarkers, it allows clinically relevant decision to be made beyond ‘maximal medical therapy and then surgery as an approach to patients with CRS (Table 1).

**Table 1:** Summary of Key Findings of CRS Phenotypes

	Phenotype		
Characteristics	CCAD (IgE mediated)	eCRS (AERD)	Non-eCRS
Clinical Presentation	- Young onset (teens to 20s) - Rhinitis symptoms - Smell preserved - Other atopic disease: o Childhood asthma o conjunctival symptoms, dermatitis	- Mid-Life “adult” onset (30–50 yo) - Occasionally post respiratory virus - “Completely well” prior to onset or if allergic, then symptoms limited to childhood - Smell loss (corticosteroid responsive) - Antibiotic seeking - Food and alcohol induced flares - Adult onset asthma linked temporally to CRS onset.	- Older onset 50 yrs.+ - Female, obese - Cough - Poor corticosteroid response - “Asthma” present but often poor response to inhaled preventive therapy (corticosteroid based)
Endoscopy	- Middle turbinate edema - Polypoid changes from turbinates and septum - No thick mucin - Normal sinus mucosa on surgery	- Polyps (small, multiple, large) from the middle meatus - Thick eosinophilic mucin - Secondary purulence	- Polyps or polypoid edema - Purulent secretions - Lack of eosinophilic mucin
Radiology	- Central thickening of septum and turbinates, peripheral clearing (CCAD) - Mucus trapping only in sinsues - Normal anterolateral sinus mucosa (“black halo”)	- Pan-sinusitis (Lund-Mackay 24) - Neo-osteogenesis	- Pan-sinusitis (undistinguishable from eCRS)
Histopathology	- Elevated tissue eosinophilia - Often without activation (no eosinophil aggregates and charcot-leyden crystals) - No serum eosinophils - Elevated total and specific IgE	- Elevated tissue eosinophilia (>10eos/hpf, but often >100eos/hpf) - Evidence of eosinophil activation (eosinophil aggregates and charcot-leyden crystals) - Serum eosinophilia	- Lack of tissue eosinophilia (< 10/HPF)
Allergy	- + allergy testing (dustmite/perennial allergens) - Often monoallergen-sensitized	- Either negative IgE sensitization or multi-allergen sensitized	- Negative skin prick, immunocap/RAST
Treatment	- Allergen directed immunotherapy - Endoscopic sinus surgery - Topical corticosteroid (spray or irrigation)	- Systemic corticosteroid treatment (up to 2–3 times per year) if limited burden of disease - Endoscopic sinus surgery (Draf 3) - Topical corticosteroid irrigations (not sprays) For AERD: - Zileuton, Montelukast, Zafirlukast - Can take selective COX-2 inhibitors (Meloxicam)	- Saline or corticosteroid irrigations - Endoscopic sinus surgery - Macrolide therapy (Clarithromycin 250 mg daily for 3 months) - Continue 3/week until 12 months if responder
Difficult to control disease	- Omaluzimab (anti-IgE)	- Mepoluzimab (anti-IL5) - Other immune-modulating therapy (Benraluzimab, Dupiliumab, Reslizumab, etc) For AERD: - ASA desensitization (1300 mg commencement and 350-700 mg daily maintenance)	- Consider re-biopsy of a patient post-surgery and post-corticosteroid based treatment if not responding and may be re-classified under this phenotype

## Data Availability

Data sharing not applicable to this article as no datasets were generated or analysed during the current study.

## Abbreviations

AERD: Aspirin exacerbated respiratory disease
AFS: Allergic fungal sinusitis
AR: Allergic rhinitis
CCAD: Central compartment atopic disease
COX: Cyclooxygenase
CRS: Chronic rhinosinusitis
CRSsNP: Chronic rhinosinusitis without nasal polyps
CRSwNP: Chronic rhinosinusitis with nasal polyps
CT: Computed tomography
eCRS: eosinophilic chronic rhinosinusitis
NSAID: Non-steroidal anti-inflammatory drugs
OMC: Ostiomeatal complex
RAST: Radioallergosorbent test
T_H_2: T-helper 2

## References

[1] Lund V, Mullol J, Fokkens W (2007). European position paper on R, nasal polyps g. European position paper on rhinosinusitis and nasal polyps 2007. Rhinol Suppl.

[2] Fokkens WJ, Lund VJ, Mullol J, et al. European position paper on rhinosinusitis and nasal polyps 2012. Rhinol Suppl. 2012;23 3 p preceding table of contents, 1-298. 22764607

[3] Wenzel SE (2012). Asthma phenotypes: the evolution from clinical to molecular approaches. Nat Med.

[4] Tomassen P, Vandeplas G, Van Zele T (2016). Inflammatory endotypes of chronic rhinosinusitis based on cluster analysis of biomarkers. J Allergy Clin Immunol.

[5] Anderson GP (2008). Endotyping asthma: new insights into key pathogenic mechanisms in a complex, heterogeneous disease. Lancet..

[6] Brunner JP, Jawad BA, McCoul ED (2017). Polypoid change of the middle turbinate and paranasal sinus polyposis are distinct entities. Otolaryngol Head Neck Surg.

[7] Wilson KF, McMains KC, Orlandi RR (2014). The association between allergy and chronic rhinosinusitis with and without nasal polyps: an evidence-based review with recommendations. Int Forum Allergy Rhinol..

[8] Hamizan AW, Loftus PA, Alvarado R (2018). Allergic phenotype of chronic rhinosinusitis based on radiologic pattern of disease. Laryngoscope..

[9] Hamizan AW, Christensen JM, Ebenzer J (2017). Middle turbinate edema as a diagnostic marker of inhalant allergy. Int Forum Allergy Rhinol..

[10] DelGaudio JM, Loftus PA, Hamizan AW, Harvey RJ, Wise SK (2017). Central compartment atopic disease. Am J Rhinol Allergy.

[11] Scadding G, Lund V (2004). Investigative rhinology.

[12] Ho J, Hamizan AW, Alvarado R, Rimmer J, Sewell WA, Harvey RJ (2018). Systemic predictors of eosinophilic chronic rhinosinusitis. Am J Rhinol Allergy..

[13] Snidvongs K, Kalish LH, Sacks R, Sivasubramaniam R, Cope D, Harvey RJ (2013). Sinus surgery and delivery method influence the effectiveness of topical corticosteroids for chronic rhinosinusitis: systeamtic review and meta-analysis. Am J Rhinol Allergy..

[14] McHugh T, Snidvongs K, Xie M, Banglawala S, Sommer D (2018). High tissue eosinophilia as a marker to predict recurrence for eosinophilic chronic rhinosinusitis: a systematic review and meta-analysis. Int Forum Allergy Rhinol..

[15] Pratt E, Collins AM, Sewell WA, Harvey RJ (2010). Antigen selection in IgE antibodies from individuals with chronic rhinosinusitis with nasal polyps. Am J Rhinol Allergy..

[16] Orgain CA, Harvey RJ (2018). The role of frontal sinus drillouts in nasal polyposis. Curr Opin Otolaryngol Head Neck Surg.

[17] Thomas WW, Harvey RJ, Rudmik L, Hwang PH, Schlosser RJ (2013). Distribution of topical agents to the paranasal sinuses: an evidence-based review with recommendations. Int Forum Allergy Rhinol..

[18] Harvey RJ, Snidvongs K, Kalish LH, Oakley GM, Sacks R (2018). Corticosteroid nasal irrigations are more effective than simple sprays in a randomized double-blinded placebo-controlled trial for chronic rhinosinusitis after sinus surgery. Int Forum Allergy Rhinol.

[19] Barham HP, Ramakrishnan VR, Knisely A (2016). Frontal sinus surgery and sinus distribution of nasal irrigation. Int Forum Allergy Rhinol..

[20] Wong E, Sansoni ER, Do TQ (2018). Cadaveric assessment of the efficacy of sinus irrigation after staged clearance of the medial maxillary wall. *Submitted to Int Forum Allergy Rhinol*.

[21] Grayson J, Cavada M, Wong E (2019). Surgical sphenoid ostial size and the effectiveness of irrigation penetration. *Accepted for poster presentation at ASOHNS*.

[22] Snidvongs K, Pratt E, Chin D, Sacks R, Earls P, Harvey RJ (2012). Corticosteroid nasal irrigations after endoscopic sinus surgery in the management of chronic rhinosinusitis. Int Forum Allergy Rhinol..

[23] Dawson B, Gutteridge I, Cervin A, Robinson D (2018). The effects of nasal lavage with betamethasone cream post-endoscopic sinus surgery: clinical trial. J Laryngol Otol.

[24] Kartush AG, Schumacher JK, Shah R, Patadia MO. Biologic agents for the treatment of chronic rhinosinusitis with nasal polyps. Am J Rhinol Allergy. 2018;1945892418814768. 10.1177/1945892418814768 30587005

[25] Lee RU, Stevenson DD (2011). Aspirin-exacerbated respiratory disease: evaluation and management. Allergy Asthma Immunol Res.

[26] Snidvongs K, Lam M, Sacks R (2012). Structured histopathology profiling of chronic rhinosinusitis in routine practice. Int Forum Allergy Rhinol..

[27] Morse JC, Li P, Ely KA, et al. Chronic rhinosinusitis in elderly patients is associated with an exaggerated neutrophilic proinflammatory response to pathogenic bacteria. J Allergy Clin Immunol. 2018. 10.1016/j.jaci.2018.10.056 PMC6408962 30468775

[28] Oakley GM, Harvey RJ, Lund VJ (2017). The role of macrolides in chronic rhinosinusitis (CRSsNP and CRSwNP). Curr Allergy Asthma Rep.

[29] Oakley GM, Christensen JM, Sacks R, Earls P, Harvey RJ (2018). Characteristics of macrolide responders in persistent post-surgical rhinosinusitis. Rhinology..

[30] Albert RK, Schuller JL, Network CCR (2014). Macrolide antibiotics and the risk of cardiac arrhythmias. Am J Respir Crit Care Med.

