# Correction to: Clinically relevant phenotypes in chronic rhinosinusitis

**DOI:** 10.1186/s40463-019-0355-6

**Published:** 2019-07-11

**Authors:** Jessica W. Grayson, Marina Cavada, Richard J. Harvey

**Affiliations:** 1 Rhinology and Skull Base Research Group, St Vincent’s Centre for Applied Medical Research, University of New South Wales, 67 Burton Street, Darlinghurst, Sydney, NSW 2010 Australia; 2 Department of Otolaryngology, Faculty of Medicine and Health Sciences, Macquarie University, Sydney, Australia

**Correction to: J Otolaryngol Head Neck Surg**

**https://doi.org/10.1186/s40463-019-0350-y**

Following publication of the original article [1], the authors reported an error in Table 1. In the second columns of the ‘Radiology’ row, ‘Normal anterolateral sinus mucosa’ should read ‘Normal superolateral sinus mucosa’. A corrected version of Table 1 is included in this Correction.

**Table 1:** Summary of Key Findings of CRS Phenotypes

	Phenotype		
Characteristics	CCAD (IgE mediated)	eCRS (AERD)	Non-eCRS
Clinical Presentation	- Young onset (teens to 20s) - Rhinitis symptoms - Smell preserved - Other atopic disease: ° Childhood asthma ° conjunctival symptoms, dermatitis	- Mid-Life “adult” onset (30–50 yo) - Occasionally post respiratory virus - “Completely well” prior to onset or if allergic, then symptoms limited to childhood - Smell loss (corticosteroid responsive) - Antibiotic seeking - Food and alcohol induced flares - Adult onset asthma linked temporally to CRS onset.	- Older onset 50 yrs.+ - Female, obese - Cough - Poor corticosteroid response - “Asthma” present but often poor response to inhaled preventive therapy (corticosteroid based)
Endoscopy	- Middle turbinate edema - Polypoid changes from turbinates and septum - No thick mucin - Normal sinus mucosa on surgery	- Polyps (small, multiple, large) from the middle meatus - Thick eosinophilic mucin - Secondary purulence	- Polyps or polypoid edema - Purulent secretions - Lack of eosinophilic mucin
Radiology	- Central thickening of septum and turbinates, peripheral clearing (CCAD) - Mucus trapping only in sinsues - Normal superolateral sinus mucosa (“black halo”)	- Pan-sinusitis (Lund-Mackay 24) - Neo-osteogenesis	- Pan-sinusitis (undistinguishable from eCRS)
Histopathology	- Elevated tissue eosinophilia - Often without activation (no eosinophil aggregates and charcot-leyden crystals) - No serum eosinophils - Elevated total and specific IgE	- Elevated tissue eosinophilia (>10eos/hpf, but often >100eos/hpf) - Evidence of eosinophil activation (eosinophil aggregates and charcot-leyden crystals) - Serum eosinophilia	- Lack of tissue eosinophilia (< 10/HPF)
Allergy	- + allergy testing (dustmite/perennial allergens) - Often monoallergen-sensitized	- Either negative IgE sensitization or multi-allergen sensitized	- Negative skin prick, immunocap/RAST
Treatment	- Allergen directed immunotherapy - Endoscopic sinus surgery - Topical corticosteroid (spray or irrigation)	- Systemic corticosteroid treatment (up to 2–3 times per year) if limited burden of disease - Endoscopic sinus surgery (Draf 3) - Topical corticosteroid irrigations (not sprays) For AERD: - Zileuton, Montelukast, Zafirlukast - Can take selective COX-2 inhibitors (Meloxicam)	- Saline or corticosteroid irrigations - Endoscopic sinus surgery - Macrolide therapy (Clarithromycin 250 mg daily for 3 months) - Continue 3/week until 12 months if responder
Difficult to control disease	- Omaluzimab (anti-IgE)	- Mepoluzimab (anti-IL5) - Other immune-modulating therapy (Benraluzimab, Dupiliumab, Reslizumab, etc) For AERD: - ASA desensitization (1300 mg commencement and 350–700 mg daily maintenance)	- Consider re-biopsy of a patient post-surgery and post-corticosteroid based treatment if not responding and may be re-classified under this phenotype
